# Supplementary material for: Imaging and controlling coherent phonon wave packets in single graphene nanoribbons
Source: Nat Commun. 2023 Jun 13;14:3484. doi: 10.1038/s41467-023-39239-1 (PMC10264436; doi:10.1038/s41467-023-39239-1)
Supplement: Supplementary file 1 — Supplementary Information [file 41467_2023_39239_MOESM1_ESM.docx]

Supplementary Information for

**Imaging and Controlling Coherent Phonon Wave Packets in Single Graphene Nanoribbons**

Yang Luo^1^, Alberto Martin-Jimenez^1^, Michele Pisarra^2^, Fernando Martin^3,4,5^, Manish Garg^1,^*, Klaus Kern^1,6^

^1^ Max Planck Institute for Solid State Research, Heisenbergstr. 1, 70569 Stuttgart, Germany

^2^ INFN-LNF, Gruppo Collegato di Cosenza, Via P. Bucci, cubo 31C, 87036, Rende (CS), Italy

^3^ Instituto Madrileño de Estudios Avanzados en Nanociencia (IMDEA Nano), Faraday 9, Cantoblanco, 28049 Madrid, Spain

^4^ Departamento de Química, Módulo 13, Universidad Autónoma de Madrid, 28049 Madrid, Spain

^5^ Condensed Matter Physics Center (IFIMAC), Universidad Autónoma de Madrid, 28049 Madrid, Spain

^6^ Institut de Physique, Ecole Polytechnique Fédérale de Lausanne, 1015 Lausanne, Switzerland

*Author to whom correspondence should be addressed. [mgarg@fkf.mpg.de](mailto:mgarg@fkf.mpg.de)

**Section I. Sample and tip preparation**

The experiments were performed in a custom-built scanning tunneling microscope (STM) operating in ultra-high vacuum conditions (~ 5×10^-10^ mbar), and at liquid nitrogen temperature (~ 90 K). Au(111) surfaces were prepared by repeated cycles of sputtering with 1.0 keV Ar^+^ ions and thermal annealing at ~ 500 °C. To fabricate graphene nanoribbons, 0.5 ML of 10,10′-dibromo-9,9′-bianthryl molecules (DBBA) were sublimated on top of the clean Au(111) sample held at room temperature. 7-armchair graphene nanoribbons (7-AGNRs) were obtained by post-annealing the sample at 400 °C for 15 minutes ^1^. Electrochemically etched Au tips were used in all the experiments to enhance the electric field confinement of the laser pulses. All topographic images presented in the current work were acquired in the ‘constant current mode’ of the STM.

All TERS spectra from the GNRs presented in the current work were measured from a single GNR in atomic point contact with the Au nanotip. To establish the atomic point contact, the Au nanotip was placed on top of the GNR extremity and the tip-sample distance was reduced by increasing the tunneling current to ~ 6 μA in the constant current mode. Atomic point contact of the GNR with the Au nanotip leads to the GNR being physically attached to the nanotip. The nonlinearity in the variation of the tunneling current on modulation of the tunnel gap distance (Δz) is significantly reduced for a GNR attached on the nanotip compared to a free nanotip as shown in Fig. S1. Measurement of such nonlinear curves confirms whether the GNR is attached on the Au nanotip or not**.** To remove the GNR from the tip, voltage pulse lasting 50 ms and set to ─2 V was used. STM images of GNRs before and after establishing the atomic point contact is shown in Fig. S2.


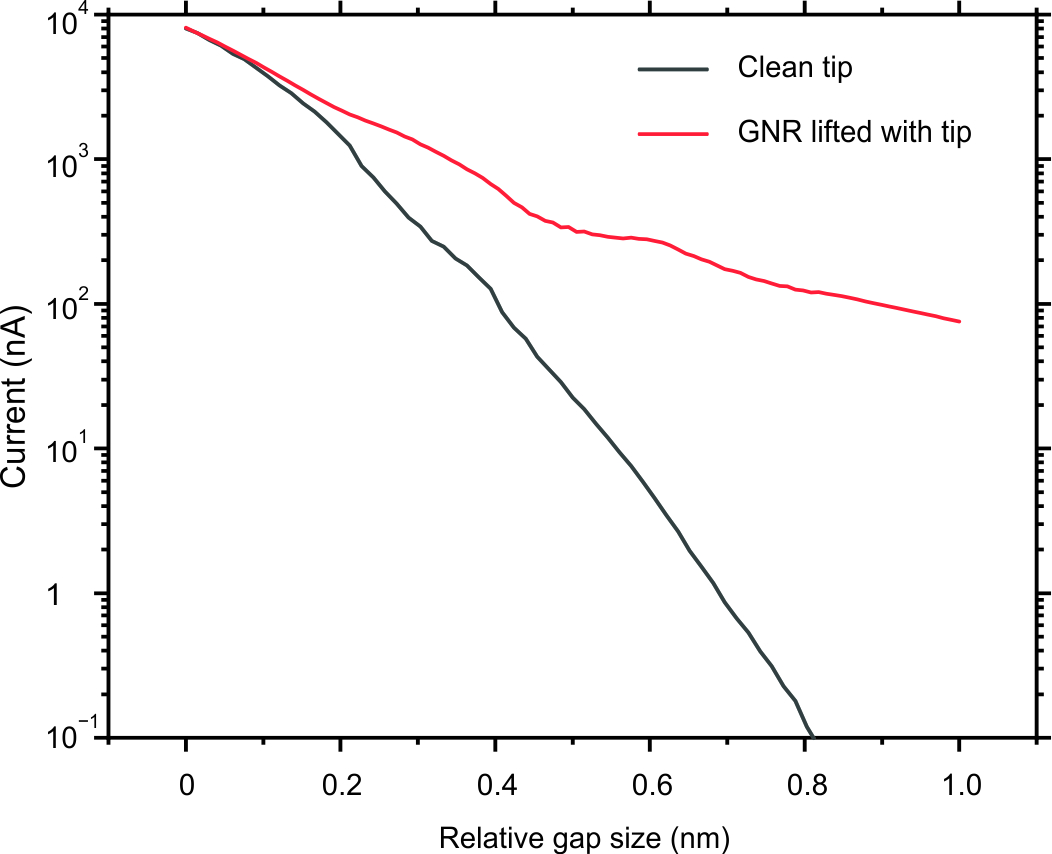


**Fig. S1 |** Comparison of the variation of the tunneling current as a function of the relative tunneling gap for a clean Au nanotip (black curve) and for an Au nanotip with a GNR attached to it (red curve). The bias voltage was set to ─500 mV.


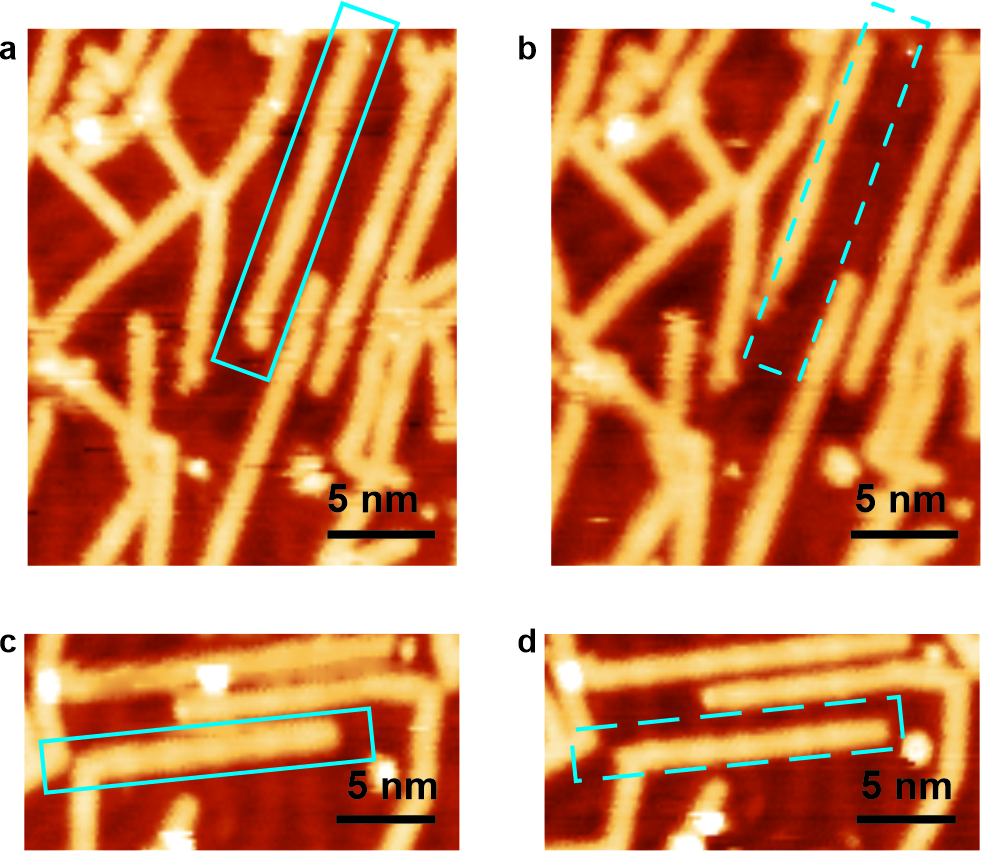


**Fig. S2 | STM images of GNRs before and after the experiments. a** and **c ,** STM image of GNRs prior to the experiments, with the measured GNR in the experiments marked by the blue rectangle. Atomic point contact was established between the Au nanotip and the GNR inside the blue rectangle for the CARS experiments. **b** and **d,** STM image of GNRs after the experiments. A voltage pulse lasting ~50 ms and set to ─2 V was used to remove the GNR from the nanotip. Before releasing the GNR, the tip was slightly shifted to the left side. The initial position of the GNR is indicated by the dashed-blue rectangle.

**Section II. Optical setup of femtosecond broadband CARS**

The optical setup of femtosecond broadband coherent anti-Stokes Raman spectroscopy (CARS) is shown in Fig. S3. The ultrafast laser system used in the current work is a Ti:Sapphire oscillator (Element™ 2, Newport Spectra-Physics) which produces laser pulses of ~ 6 fs duration with a bandwidth spanning from 650 nm to 1050 nm at a repetition rate of ~ 80 MHz. Probe pulses centered at ~ 728 nm with a duration of ~ 500 fs were generated by narrowband filtering (Ultra Narrow Bandpass Filter 728.1/1.5, AHF) of the broadband ~ 6 fs long laser pulses. Pump pulses (~ 750 - 805 nm) and Stokes pulses (~ 805 - 920 nm) were also generated by bandpass filtering of the broadband laser pulses. Two precise (resolution ~ 0.1 μm) delay stages were used to control the delay time τ_12_ between the pump and Stokes pulses and τ_23_ between the Stokes and probe pulses. An achromatic lens (diameter: 50 mm; focusing length: 75 mm) was mounted inside the UHV chamber to focus the laser beams onto the apex of the Au tip. The TERS signal was collected through the same achromatic lens and then focused onto the entrance slit of a spectrometer (Kymera 328i, ANDOR) and detected by a thermoelectrically cooled charge coupled device (iDus 416, ANDOR). TERS experiments were also performed with CW excitation by using a Helium-Neon (He-Ne) CW laser (HNL150L, Thorlabs) centered at ~ 633 nm.


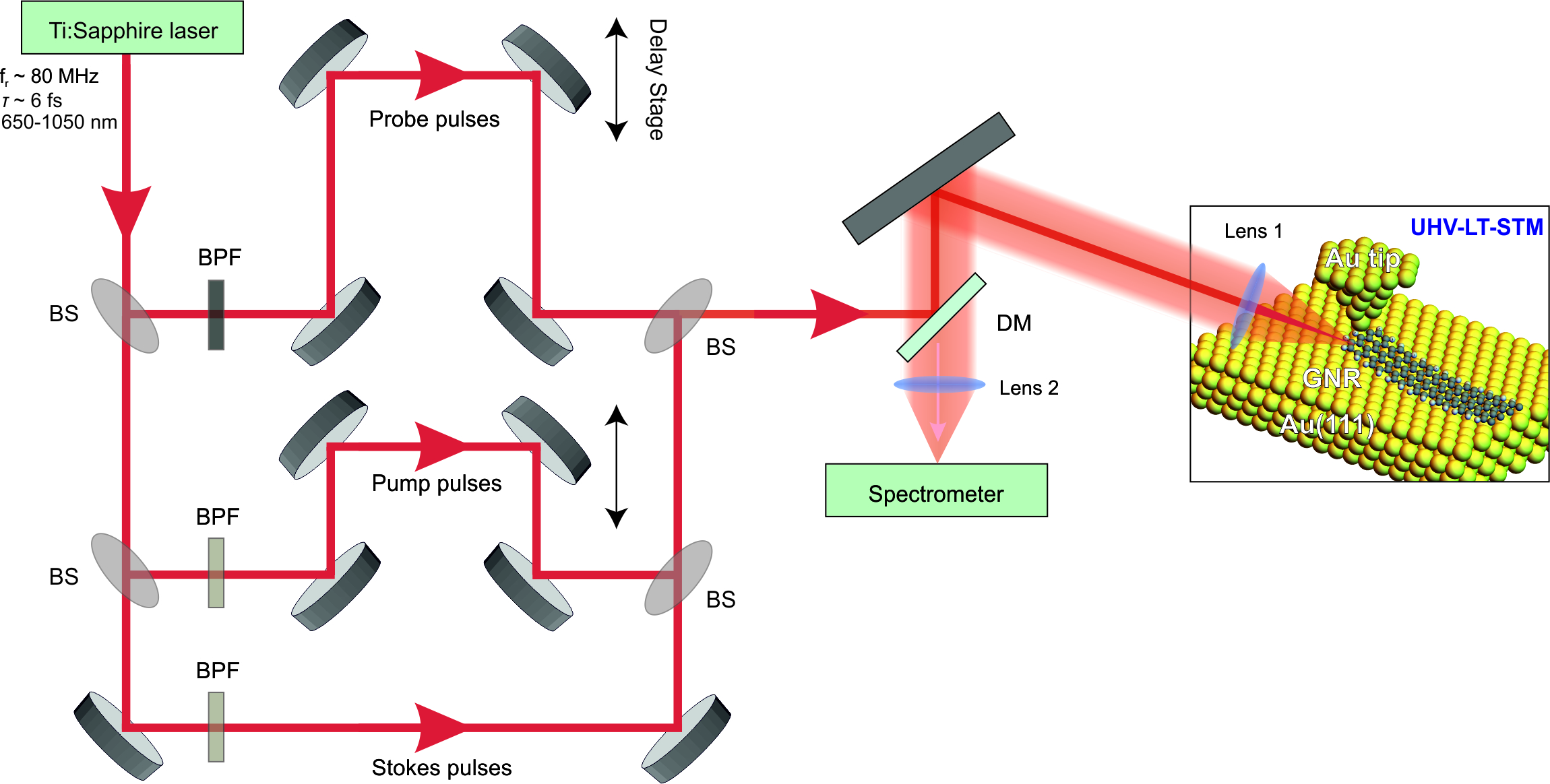


**Fig. S3 | Optical setup of broadband CARS.** BS: beam splitter; BPF: bandpass filter; DM: dichroic mirror; DM: dichroic mirror.

**Section III. Power dependence of the spontaneous (incoherent) Stokes and anti-Stokes Raman spectra**


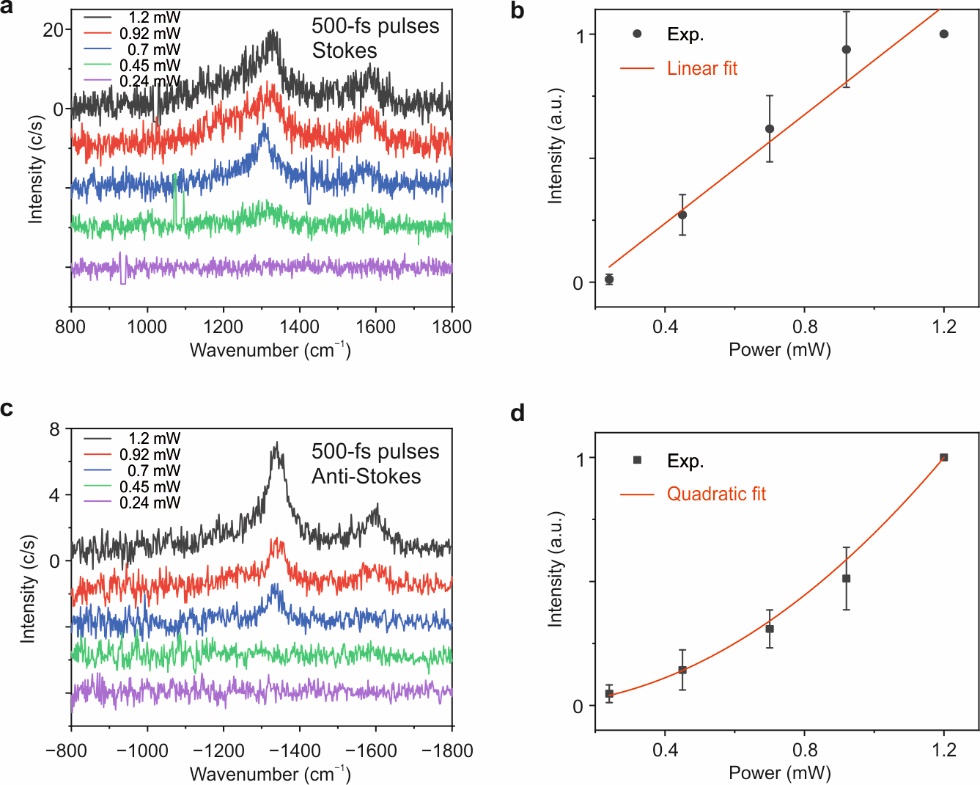


**Fig. S4 | Power dependence of the spontaneous (incoherent) Stokes and anti-Stokes Raman spectra of a GNR. a**, A series of Stokes Raman spectra measured as a function of the increasing power of the incident ultrashort probe pulses (~ 500 fs long) in the range from 0.24 to 1.2 mW. The bias voltage (*V*) and tunneling current (*I*) in the STM junction were ─500 mV and 8 µA, respectively. **b,** Scaling of the overall TERS signal in **a** as a function of the increasing power of the exciting ultrashort laser pulses. **c**, A series of anti-Stokes Raman spectra measured as a function of the increasing power of the incident probe pulses in power range from 0.24 to 1.2 mW (*V* = ─500 mV, *I* = 8 µA). **d**, Scaling of the overall anti-Stokes TERS signal in **c** as a function of the increasing power of the exciting ultrashort laser pulses. Red-curves in **b** and **d** represent the linear and quadratic fits of the power-dependent scaling of the Stokes and anti-Stokes TERS signals, respectively.

**Section IV. Population decay time (T_1_) of phonons in a single GNR**


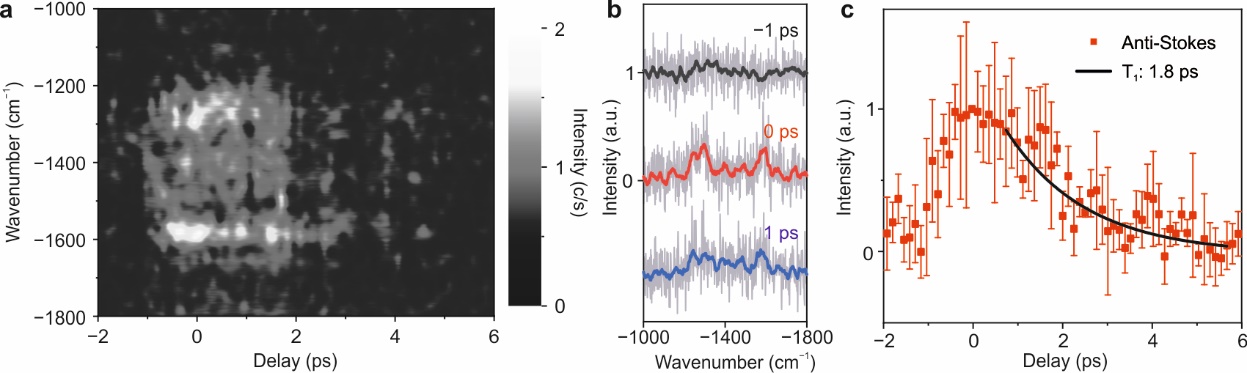


**Fig. S5 | Population relaxation dynamics of phonons in a single GNR. a,** Population relaxation dynamics of phonons in an excited GNR was studied by time-resolved incoherent anti-Stokes Raman spectroscopy (IARS). Here, the pump pulse (~ 750-805 nm, ~ 100 fs long) was used to excite the phonons from the ground to excited states in a GNR, and the delayed probe pulse (728±1.5 nm, ~ 500 fs long) was used to track the evolution of phonon population in different states by anti-Stokes Raman scattering, thereby revealing the population decay dynamics of the phonons. A series of IARS spectra were measured as a function of the delay between pump and probe pulses. Measurement parameters: *P*_pump_ = 2.0 mW, *P*_probe_ = 1.0 mW, *V* = ─500 mV, *I* = 4 µA. **b,** Anti-Stokes Raman spectra (IARS) of a GNR acquired at three relative delays between the pump and probe laser pulses from the measurement in **a.** The relative delays between the pulses are annotated on top of the spectra**. c,** Spectral integration of the Raman peaks from the individual spectral measurements in **a** as a function of the relative delay between pump and probe laser pulses (red points). Positive delay refers to the situation when the probe pulse comes after the pump pulse. An exponential fitting (black-curve) of the measurement reveals a phonon population decay time (T_1_) to be approximately 1.8 ps for a single GNR.

**Section V. Time-resolved broadband CARS of single-walled carbon nanotubes**

In our experiments, we track ultrafast phonon oscillations (coherences) by generating an impulsively excited vibrational wave packet in an interacting system by two delay-controlled (varying) broadband ultrashort pulses (pump and Stokes) and tracking the delay dependent population in the vibrational states with a much longer probe pulse. In order to substantiate our approach, we performed experiments by using the same pulse parameters as in the TERS measurements (main-text), this time on an ensemble of single-walled carbon nanotubes (SWCNTs) in ambient conditions. Commercially available SWCNTs (Sigma-Aldrich) were dispersed in dichloroethane and drop-casted on a glass film and illuminated with pulse-sequence as described in the main-text. The experimental set-up is shown in Fig. S6a. An anti-Stokes Raman spectrum of the SWCNTs was measured on exposure of the sample to only the probe pulse centered at ~728 nm with a bandwidth of ~ 1.5 nm as shown in Fig. S6b. The G mode at ~1590 cm^-1^ and D-like mode at ~1310 cm^-1^ can be identified. Fig. S6c shows a series of CARS spectra measured as a function of the delay with the probe pulses (τ_23_). Here, pump and Stokes pulses temporally overlap (τ_12_ = 0). In contrast to anti-Stokes scattering generated by only probe pulses (Fig. S6b), the CARS spectrum exhibits the vibrational peaks at 1310 cm^-1^ (D1 band), 1590 cm^-1^ (G band) and a pronounced additional peak around ~ 1440 cm^-1^ (D2 band). The origin of this peak is related to the splitting of the D band in SWCNTs ^2,3^.


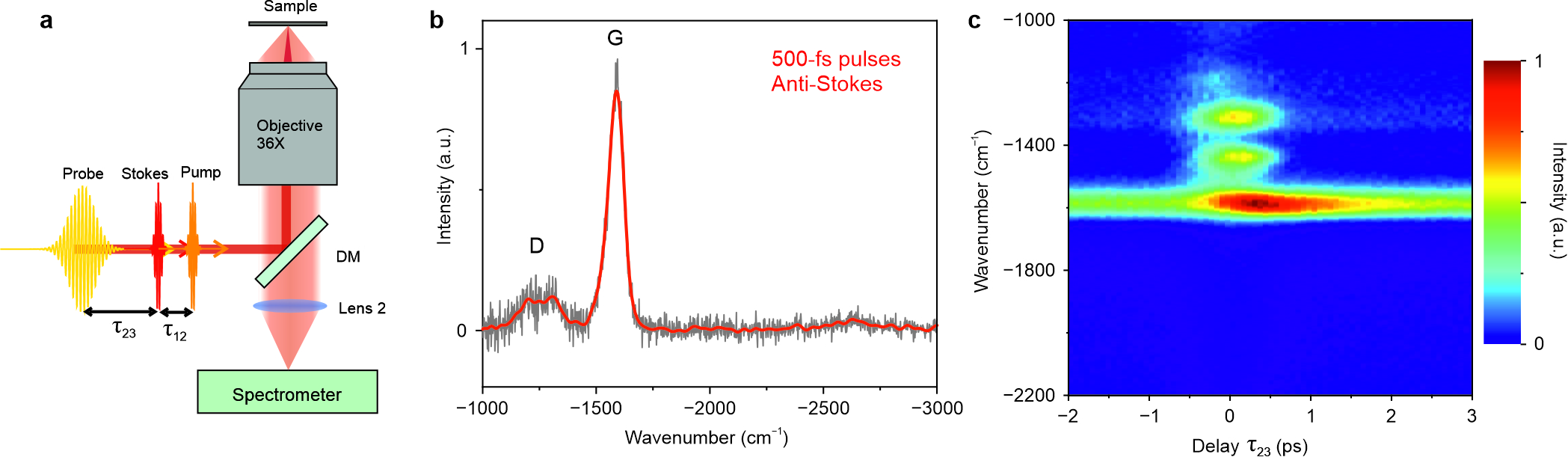


**Fig. S6 | Time-resolved broadband CARS on single-walled carbon nanotubes (SWCNTs). a,** Experimental set-up of time-resolved CARS experiments performed on SWCNTs drop casted on a glass film. A 36× objective is used to focus the laser pulses onto the sample as well as to collect the Raman signal generated from CNTs. **b,** Anti-Stokes Raman spectrum acquired on excitation with ~ 500 fs long laser pulses (*λ* = 728 nm, *P* = 0.93 mW). **c,** A series of time-resolved CARS spectra of SWCNTs acquired at various delays τ_23_ between the probe pulse and the pump and Stokes pulses (*P*_pump_ = 1.4 mW, *P*_Stokes_ = 1.2 mW, *P*_probe_ = 1.3 mW, acquisition time is 1 s). The pump and Stokes pulses overlap in time (τ_12_ = 0).

Coherent oscillations of phonons in SWCNTs were tracked following the approach described in the main text (Fig. 4). In this case, Stokes and probe pulses overlap in time (τ_23_ = 0) and the delay of the pump pulse with respect to the former pulses (τ_12_) is varied. Anti-Stokes spectra recorded as a function of the τ_12_ delay is shown in Fig. S7a. Spectral shift of the Raman bands due to positive chirp of the pump and Stokes as discussed in the main-text can also be seen in the measurements on SWCNT as shown in Fig. S7a. Temporal evolution of the spectral intensity of D1 (~ 1310 cm^-1^) and G bands (~ 1590 cm^-1^) as a function of the τ_12_ delay from the measurement in Fig. S7a is shown in Fig. S7b. To understand the origin of the oscillations in the spectral intensity of the Raman peaks in Fig. S7a, we perform Fourier transformations of the spectral intensity oscillations at all spectral points (y-axis), see Fig. S7a. A two-dimensional map of quantum beatings of phonons (coherences) is shown in Fig. S7c. The amplitude of the Fourier transformation is plotted as a function of the wavenumber of the anti-Stokes scattering (y-axis) and the frequency of the quantum beatings (x-axis), which is obtained from the experimental delay-axis (τ_12_). A cross-cut of the 2D map at the frequency of the phonon modes at ~1310 cm^─1^ and ~1590 cm^─1^ is shown in Fig. S7d.

Plotting the 2D quantum beat map with an equal range in the spectral as well as in the beating axis as in Fig. S7d provides a direct access to the states responsible for the existence of a particular beating frequency in a Raman band; the spectral axis is plotted from ─ 1150 to ─1650 cm^-1^ whereas the beating-axis is plotted from 0 to 500 cm^-1^. Plotting straight lines at 45° (negative slope) through all the beating frequencies for a particular band (e.g. G band) and marking the Raman band where the line intercepts the y-axis enables a correlation of the origin of the quantum beat. For example, the green-dashed curve passing through the beating frequency at ~ 280 cm^-1^ intercepts the y-axis at the frequency corresponding to the D1 band, whereas the orange-dashed curve passing through the beating frequency at ~ 170 cm^-1^ intercepts at the D2 band. The energy separation of the two bands with respect to the G band is justified by the observed beating frequencies. If the phonon beating frequency of ~ 280 cm^-1^ corresponding to a beating between D1 and G bands is present for the spectral intensity oscillations of the G band, counterintuitively, the same beating frequency should also be present in the spectral intensity oscillations of the D1 band. This is precisely the case as shown in Fig. S7c. The beating frequency of ~ 170 cm^-1^ in the G band (red-curve in Fig. S7d) corresponds to the phonon beating between the G band and the D2 band at ~ 1440 cm^-1^. Similarly, a beating frequency of ~140 cm^-1^ is measured in the spectral intensity oscillation in the D1 band, which is due to the beating between the D1 band and the D2 band. The slight deviation between the measured beating frequencies and the energy separation of the involved vibrational states (< 20 cm^-1^) is related to the coherent tuning of the position of the vibrational peaks due to the chirped nature of pump and Stokes pulses.


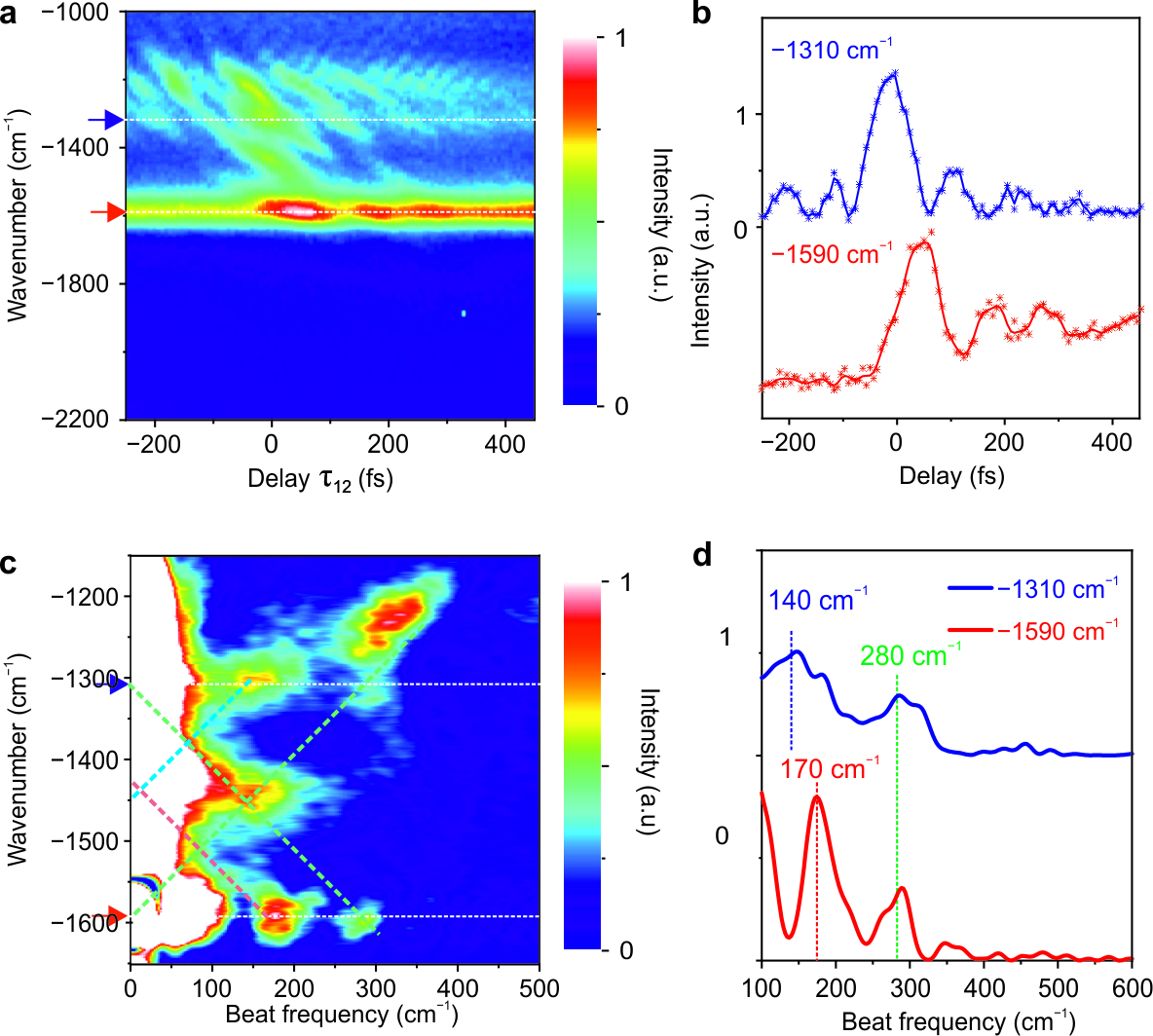


**Fig. S7 | Tracking Ultrafast Coherent Oscillation of Phonons in CNTs. a,** A series of anti-Stokes spectra acquired by delaying the pump pulse with respect to the probe and Stokes pulses. The probe and Stokes pulses overlap in time (modulation of τ_12_, τ_23_ = 0, *P*_pump_ = 1.4 mW, *P*_Stokes_ = 1.2 mW, *P*_probe_ = 1.3 mW, acquisition time is 1 s). **b,** Temporal variation of the spectral intensities of two different phonon modes at 1310 cm^─1^ and 1590 cm^─1^ from the measurement shown in **a** as a function of the delay between pump and Stokes pulses (modulation of τ_12_, τ_23_ = 0). The two phonon modes are also indicated by corresponding colour coded arrows in **a. c,** A two-dimensional map of quantum beatings of phonon modes obtained by Fourier transformation of the temporal modulations at all points in the spectral-axis in the anti-Stokes spectra shown in **a**. The amplitude of the Fourier transform is plotted as a function of the beating frequencies (x-axis) and the wavenumber of the anti-Stokes scattering (y-axis). **d**, A cross-cut of the two-dimensional quantum beat map in **c** at the frequency of the phonon modes at ~1310 cm^─1^ and ~1590 cm^─1^.

**Section VI. Time-resolved broadband CARS of graphite**

In order to elucidate how pulse chirping impacts quantum beating, we also performed a control experiment where we track vibrational coherences in a graphite sample by systematic variation of the linear chirp of the ultrashort pump and Stokes pulses. The experiments were performed for four different positive chirps of the pulses. The chirps of the pulses were tuned by the application of three pairs of chirped dielectric mirrors (CDM). The group delay dispersion (GDD) of the CDM is ~ -40 fs^2^ for one pair of reflections off the CDM. In order to make our pulses nearly chirp free, the pulses had to be reflected 72 times off the CDM, this significantly reduces the power of the pulses by nearly half. This leads to a dramatic reduction of the CARS in GNRs and carbon nanotubes (CNTs), as CARS is a nonlinear four wave mixing process. Therefore, in order to compensate for this reduction in the power of the pulses in going from positively chirped pulses to nearly chirp free pulses, a more nonlinear bulk media was chosen, namely, highly oriented pyrolytic graphite (HOPG).

Fig. S8 below shows the experimental setup and the measurement of CARS signal in the graphite sample. To achieve better control over the chirped pulses, we have slightly reduced the bandwidth of the Stokes pulses. Fig. S9 shows the measurement of vibrational coherences between D and G bands of graphite for different positive chirps of the pump and Stokes pulses. The measurement in Fig. S9a is for nearly chirp free pump and Stokes pulses, whereas, for the measurements shown in Fig. S9b to Fig. S9d, a positive chirp was tuned gradually by reducing the number of reflections off the CDM of the pulses. Fig. S9e to Fig. S9h show the oscillations in the spectral intensity of D (~ -1400 cm^-1^) and G (~ -1580cm^-1^) bands of graphite for four different cases of positive chirps of the pulses as shown in Fig.S9a-d. For the case of nearly chirp free pulses, the spectral intensities of the D and G bands (Fig. S9e) evolve completely out of phase, as it would be the case for quantum beating between two levels. The frequency of these oscillations, obtained by FFT, matches nearly perfectly with the energy difference between the D and G bands as shown in Fig. S9i. In the case of positively chirped pump and Stokes pulses (Fig. S9f to Fig. S9h), the time period of oscillations remains exactly the same as for the nearly chirp free pulses (Fig. S9e). However, due to spectral shifts of the D and G bands on application of chirped pump and Stokes pulses (Fig. S9b to Fig. S9d), the relative phase between the spectral intensity oscillations of the D and G bands changes (Fig. S9f to Fig. S9h). Nevertheless, the observed frequency of the vibrational coherences for the case of chirped pump and Stokes pulses (Fig.S9j to Fig. S9l) remain exactly the same as for the case of nearly chirp free pulses (Fig. S9i).

As we mentioned earlier, multiple reflections off the CDM to get chirp free pulses in the STM junction reduces the power of the pulses used in the experiments by nearly half. This makes the experiment with nearly chirp free pulses in the GNR experiment very difficult due to nearly 4 to 8 times weaker CARS signal level as shown by power scaling measurements in Fig. 2d. However, we have now clearly shown, by the systematic control experiment in the graphite sample, that the frequency of the measured vibrational coherences remains the same for chirp free and positively chirped pump and Stokes pulses. Moreover, the use of positively chirped pulses provides an additional control in the measurements and shows the capability of spectrally shifting the positions of the Raman peaks.


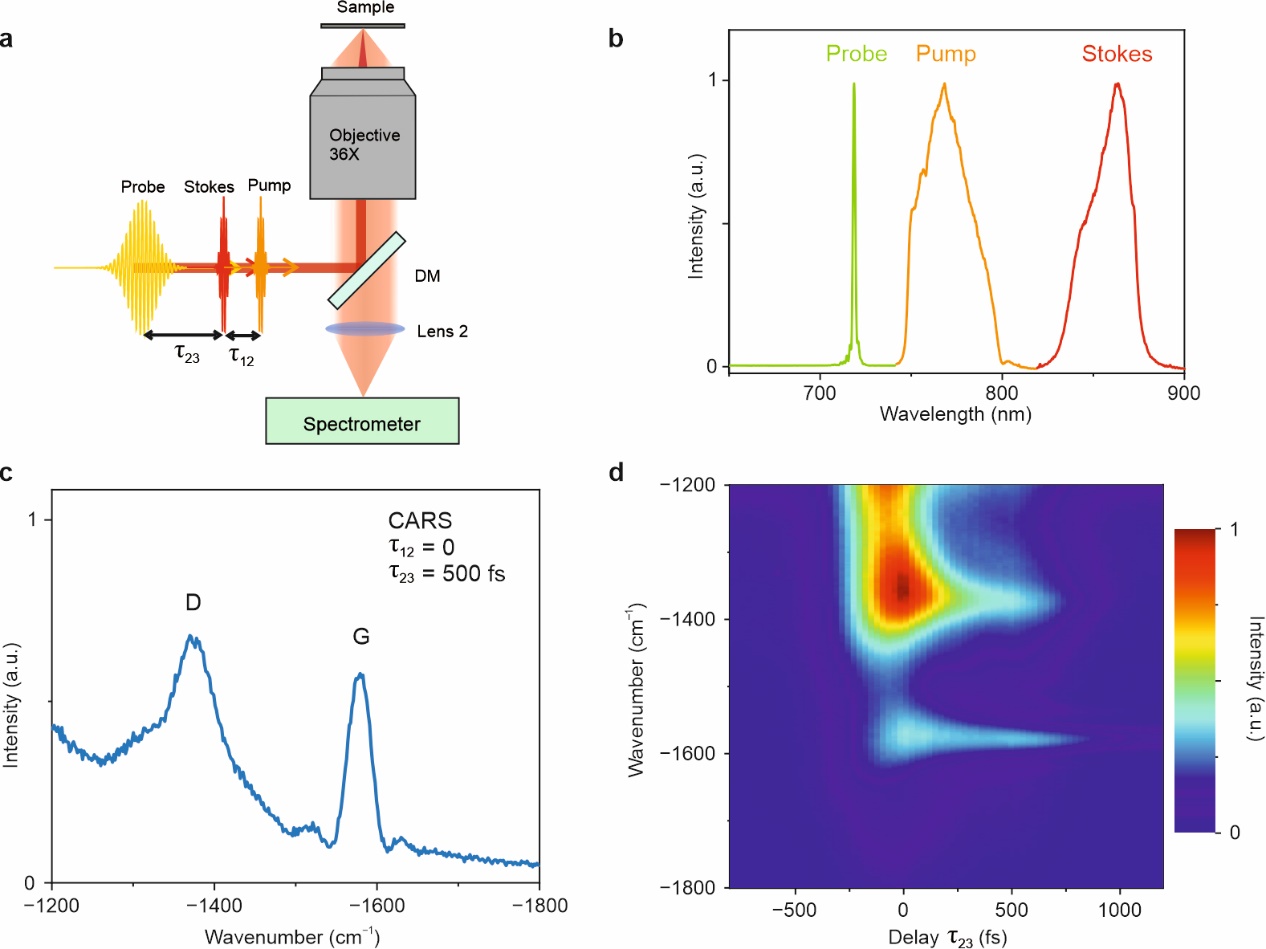


**Fig. S8 | Time-resolved broadband CARS in highly oriented pyrolytic graphite (HOPG). a,** Experimental set-up of time-resolved CARS experiments performed on highly oriented pyrolytic graphite (HOPG). A 36× objective is used to focus the laser pulses onto the sample as well as to collect the Raman signal generated from HOPG. **b,** Spectra of pump, probe and Stokes pulses as used in the experiment. **c,** A coherent anti-Stokes Raman spectrum (CARS) acquired at a relative delay of ~ 500 fs between the pump/Stokes and the probe pulses. **d,** A series of time-resolved CARS spectra of HOPG acquired at various delays τ_23_ between the probe pulse and the pump and Stokes pulses (*P*_pump_ = 1.5 mW, *P*_Stokes_ = 0.9 mW, *P*_probe_ = 0.6 mW, acquisition time is 5 s). The pump and Stokes pulses overlap in time (τ_12_ = 0).


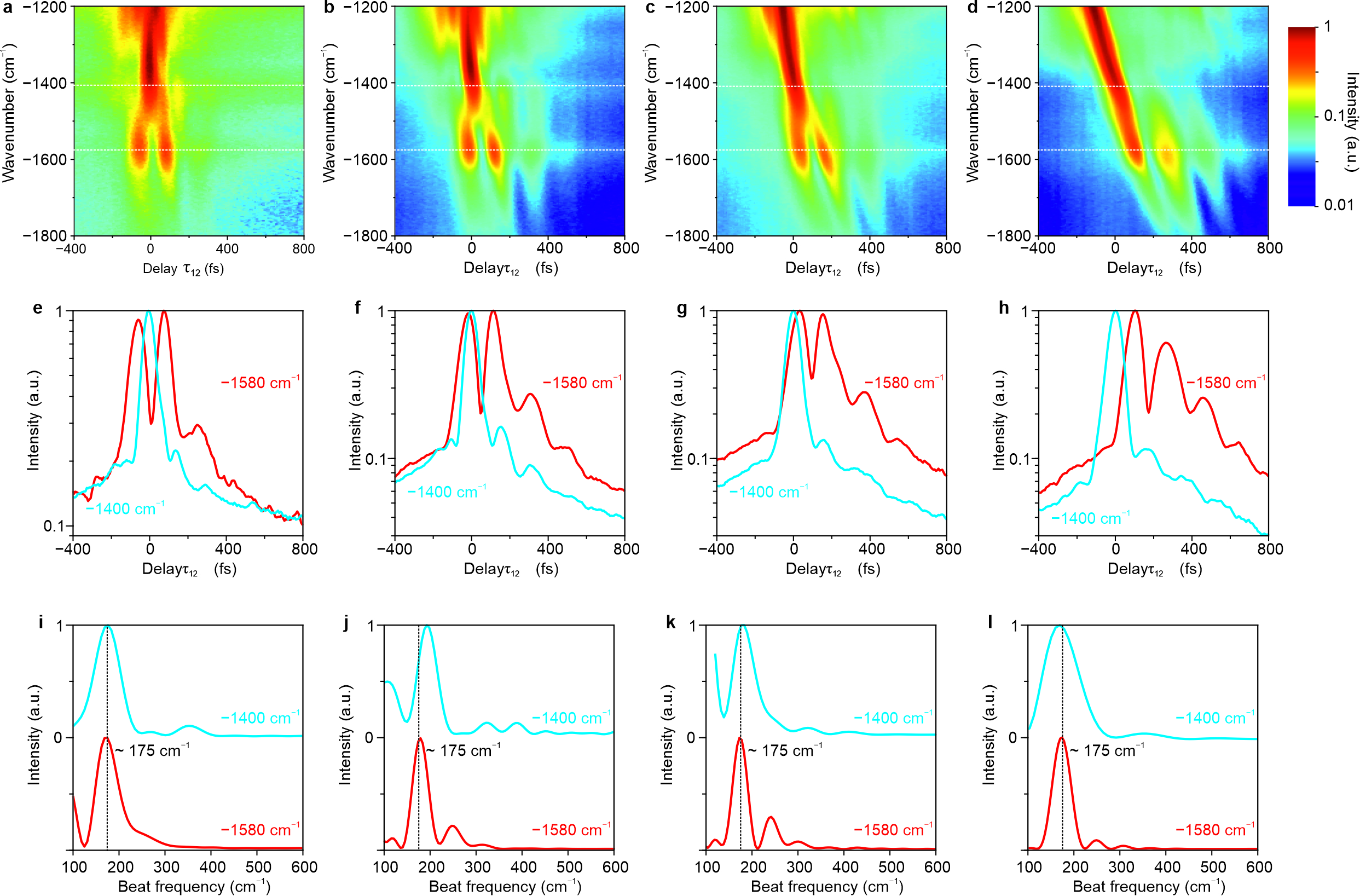


**Fig. S9 | Tracking Ultrafast Coherent Oscillation of Phonons in Graphite. a, b, c** and **d,** A series of anti-Stokes spectra acquired by delaying the pump pulse with respect to the probe and Stokes pulses. The pump and Stokes pulses in **a** are nearly chirp free. The chirp free pump and Stokes pulses were obtained by 36 pairs of reflection off three pairs of chirped dielectric mirrors (CDM). Measurements in **b, c,** and **d** were done with positively chirped pump and Stokes pulses obtained by 26 pairs of reflections, 14 pairs of reflections and no reflections, respectively off the CDM. The probe and Stokes pulses overlap in time (modulation of τ_12_, τ_23_ = 0, acquisition time is 1 s) for measurements shown in **a, b, c** and **d**. **e, f, g,** and **h,** Temporal variation of the spectral intensities of two different phonon modes at around -1400 cm^─1^ (D-band) and -1580 cm^─1^ (G-band) from the measurement shown in **a, b, c** and **d,** respectively**,** as a function of the delay between pump and Stokes pulses (modulation of τ_12_, τ_23_ = 0). The two phonon modes are also indicated by dashed white lines in **a, b, c** and **d. i, j, k** and **l,** Frequency spectrum of oscillations of spectral intensities of D and G bands of graphite shown in **e, f, g** and **h,** respectively.

**Section VII. Frequency-resolved optical-gating characterization of Stokes and pump laser pulses.**

We conducted the temporal characterization of the pulses as used in the experiment using second-harmonic frequency-resolved optical gating (FROG) measurements. The experimental FROG traces and the reconstructed FROG traces of the Stokes (805-950 nm) and pump (750-805 nm) pulses are shown below in Fig. S10. FROG measurements of the compressed Stokes and pump pulses are shown in Fig. S10a and S10c, respectively. The pulse durations (τ) were determined at the intensity levels of 1/e^2^. The phase profiles are nearly flat in both the temporal and spectral domains, indicating that the pulses were effectively compressed (chirp free). The laser pulses traversed through several optical lenses and an optical window, resulting in an estimated total length of ~ 3.5 cm glass in the beam path, prior to reaching the STM junction. Therefore, we added a glass of 3.5 cm thickness in the beam path of the FROG measurements to estimate the pulse shape at the STM junction and measure the temporal profile of the chirped pulses. FROG measurements of the chirped Stokes and pump pulses are shown in Fig. S10b and S10d. A noticeable increase in the pulse durations is apparent when compared to the compressed (chirp free) pulses. The phase profiles display a quadratic behavior, indicating the presence of a second-order spectral phase. This suggests that the laser pulses are stretched by a linear chirp rather than nonlinear chirps.


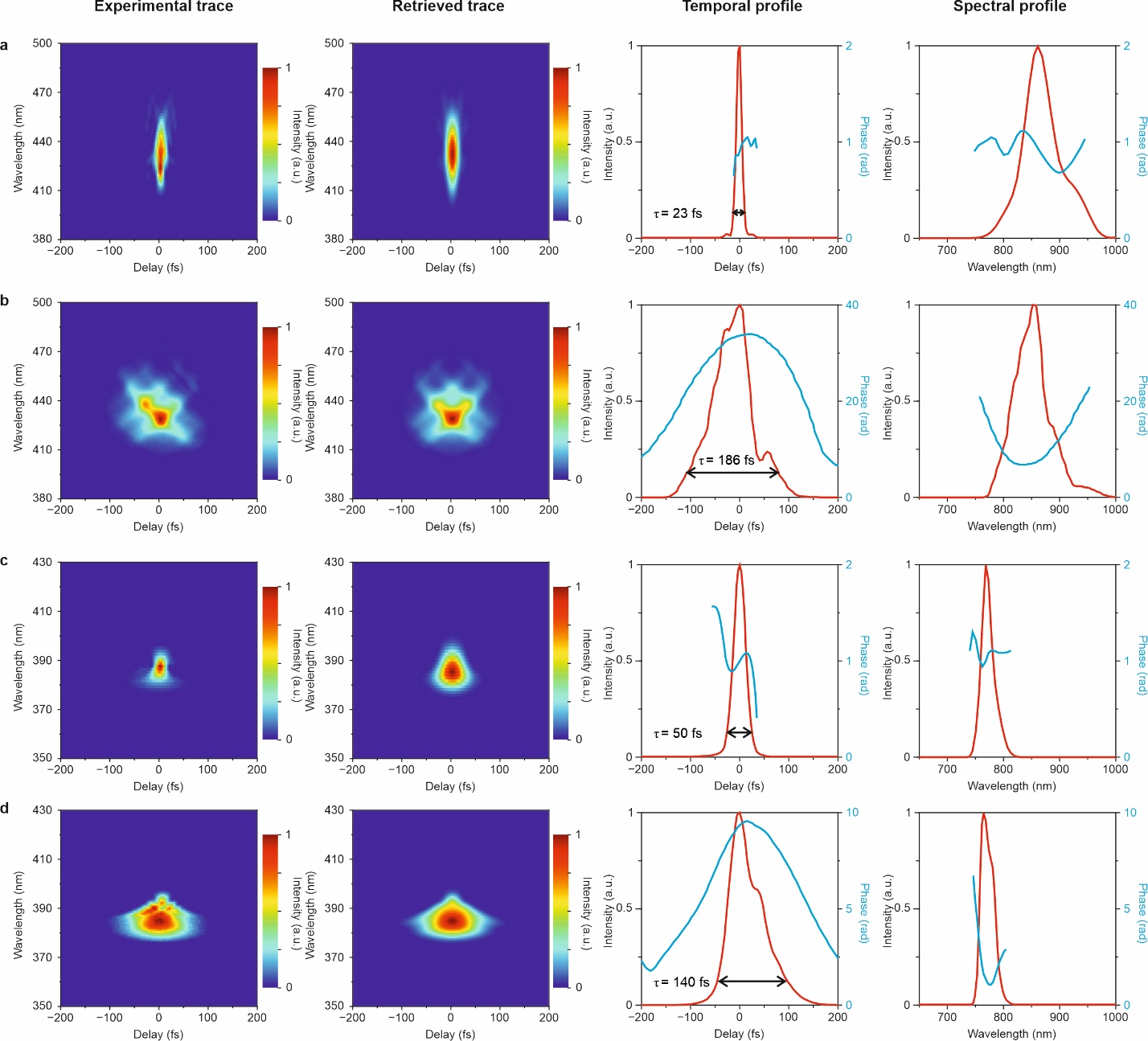


**Fig. S10 | Frequency-resolved optical-gating measurements of Stokes and pump laser pulses.** First Column: The experimental FROG traces. Second Column: The reconstructed FROG traces. Third Column: The temporal profiles of laser intensity (red-curve) and phase (blue-curve). Fourth Column: The spectral profiles of laser intensity (red-curve) and phase (blue-curve). FROG measurements and reconstructions for compressed (chirp free) Stokes pulse (**a**), the chirped Stokes pulse (**b**), the compressed (chirp free) Pump pulse (**c**), and the chirped Pump pulse (**d**). The pulse durations τ are determined at the intensity levels of 1/e^2^.

**Section VIII. Density Functional Theory Calculations**

**VIII.1 Technical details**

Density Functional Theory (DFT) calculations were carried out within the Projector Augmented Wave (PAW) method ^4^ as implemented in the VASP code ^5-7^ using the PBE exchange-correlation functional ^8^. In all calculations, the plane waves cut-off energy was set to 400 eV, whereas the 1^st^ Brillouin Zone sampling was limited to the Γ point. The DFT geometry optimizations of the graphene nanoribbons object of this study (see below) were run adopting a tight 10^-6^ eV convergence criterion for the self-consistent cycles, optimizing the position of the mobile atoms until the maximum residual force was lower than 10^-3^ eV/A. With the equilibrium configurations at hand, the vibrational normal modes were determined by computing the dynamical matrix with a finite difference approach with a very tight 10^-8^ eV convergence criterion for the self-consistent cycles. The Raman intensities for each normal mode in the 950-1650cm^-1^ window were determined via the variation of the macroscopic static dielectric tensor ^9^ as computed by VASP ^10^, employing the method implemented by A. Fonari and S. Stauffer ^11^.

**VIII.2 Geometry information**

In this work we analyzed armchair graphene nanoribbons characterized by 7 C2 dimers across the ribbon width (7-AGNR). We constructed the finite size ribbons by repeating the 7-AGNR unit cell (green shaded rectangle in Fig. S11a) 5, 6, and 7 times to obtain the L5, L6, and L7 geometries, saturating the dangling bonds at the terminations with H atoms. The coordinates of all the atoms were then optimized to obtain the equilibrium length of the “flat” configurations of the finite size nanoribbons. The curved geometries were then obtained by rolling the ribbon on the long side at the desired angle α keeping the total length constant (see the construction in Fig. S11b for the meaning of the bending angle). After this construction, the geometry was optimized again, freezing the atoms at the extrema (the atoms shown with a different color in Fig. S11b) so that the curved geometry is kept; in this way, we mimic the relatively strong interaction of the ribbon extrema with the gold surface and nanotip. In this work we adopted angles of 2°, 5°, and 10°, which result in vertical displacements of the atoms of the same order of the $\Delta z$ gap measured in the STM junction. In particular, we point out that for the L7 ribbon with $\alpha=10^{\circ}$ a maximum $\Delta z\sim2.5Å$ was obtained (see also Fig. S12, where we report the optimized geometries of all the structures analyzed in this work).


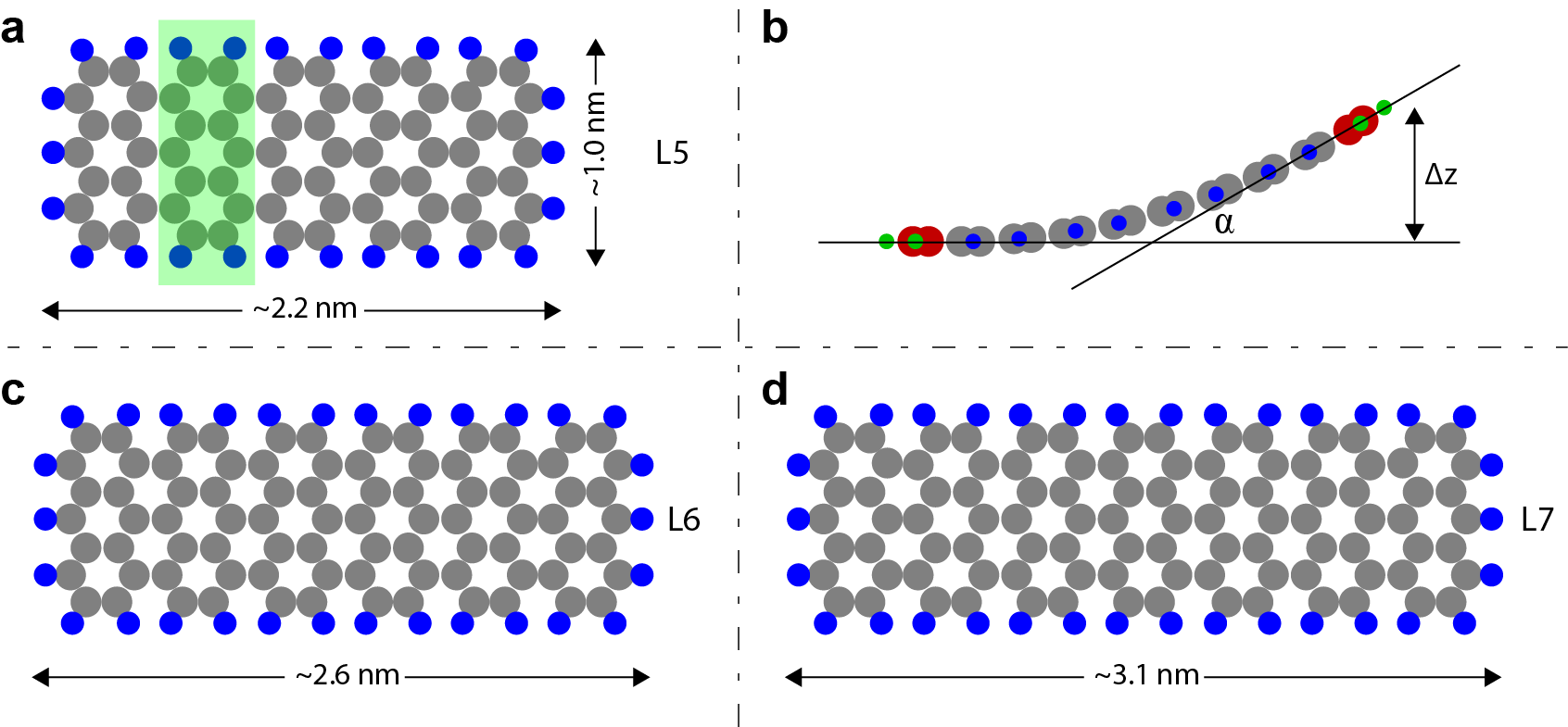


**Fig. S11 | Geometry information I. a,** Top view of the L5 graphene nanoribbon in the flat geometry; the gray circles mark the C atoms, the blue circles the H atoms; the green shaded area includes the atoms inside a 7-AGNR unit cell. **b,** Construction of the rolling angle α for the L5 nanoribbon: a side view of the ribbon is shown; the atoms appearing in red/green are kept fixed in the optimization of the curved geometry; the value of the angle α has been exaggerated for clarity. **c, d,** Top views of the L6 and L7 graphene nanoribbon in the flat geometry.


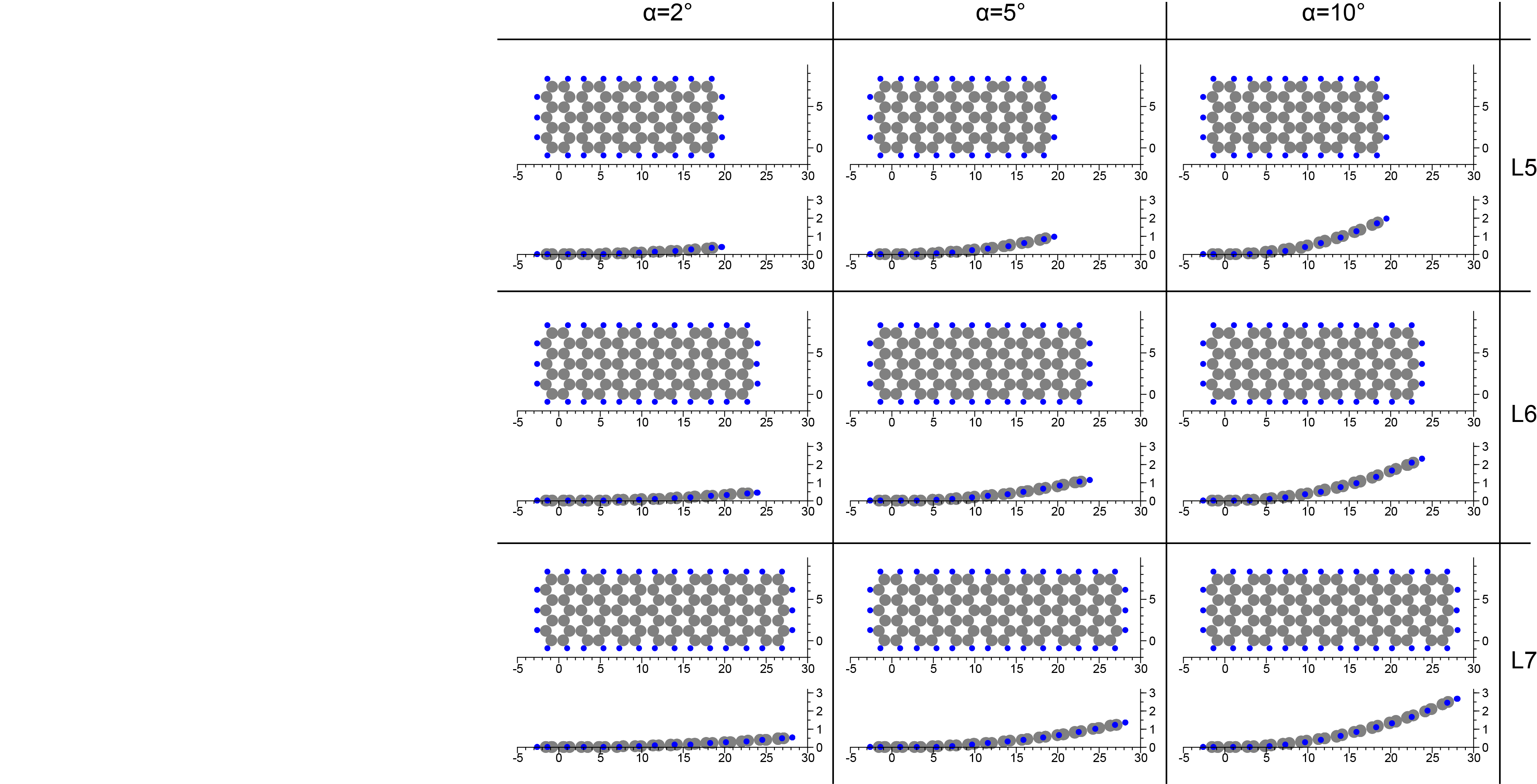


**Fig. S12 | Geometry information II.** Top views and side views of the equilibrium geometries for all the nanoribbons analyzed in this work. All the quantities reported in the figures are in Angstroms; the vertical scale in the side views has been magnified by a factor of 10; in all panels we have used the same scale and plotted range to ease the comparison.

**VIII.3 Raman Spectra**

In Fig. S13 we show the calculated Raman spectra in the 950-1650 cm^-1^ frequency range for self-standing L5, L6, and L7 curved graphene nanoribbons for three different values of α. All the spectra are characterized by several peaks. It is worth noting that, for a given nanoribbon, the spectra barely change with the angle α. Also, the spectra are qualitatively similar for the three considered sizes. In all cases, the Raman spectrum shows small peaks below 1200 cm^-1^ and then two intense peaks in the 1250-1350 cm^-1^ range, with several less intense peaks. At ~1570 cm^-1^, all spectra exhibit a very intense peak. A detailed analysis of the normal modes with the highest Raman activity is given in Fig. S14, where we show the atom displacements of the normal modes that generate the three most intense peaks of the calculated Raman spectrum for the L6 structure at $\alpha=2^{\circ}$. As it is clear from this figure, the peak at ~1270 cm^-1^ is generated by a normal mode with the largest atom displacements on the edge of the ribbon. The peak at ~1330 cm^-1^ is generated by two Raman-active vibrations in which the displacements involve both the central atoms and the edge atoms, and are directed both in the parallel and in the perpendicular directions to the long side of the ribbon. Finally, the peak at ~1570 cm^-1^ is generated by a normal mode which involves mostly the central atoms of the ribbon, which move in the direction parallel to the long side of the ribbon. It is worth mentioning that similar results are obtained, regardless of the angle α, for all three analyzed lengths. We point out that the increasing trend in the intensity of the Raman peaks at ~1330 cm^-1^ and ~1570 cm^-1^ with the length of the ribbon is consistent with the nature of the normal mode displacements parallel to the long side of the ribbon.


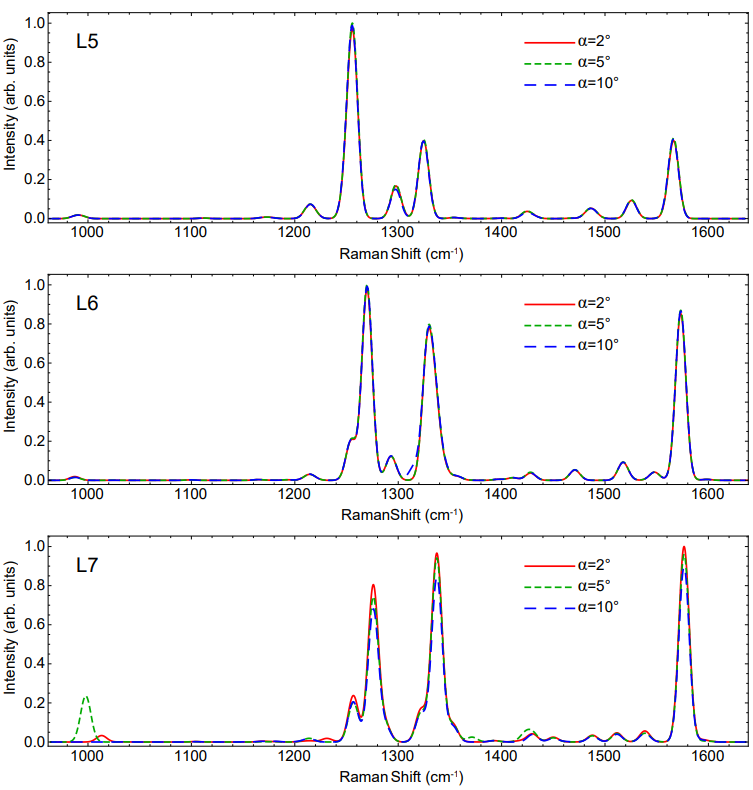


**Fig. S13 | Variation of the Raman spectra with bending angle and ribbon length.** Calculated Raman spectra for the L5(top), L6(middle), and L7(bottom) finite size graphene nanoribbons for bending angles 2°, 5°, and 10°. The spectra are obtained convoluting the calculated Raman activity with a gaussian function (standard deviation σ=5cm^-1^). The spectra are normalized to ease comparison.

**
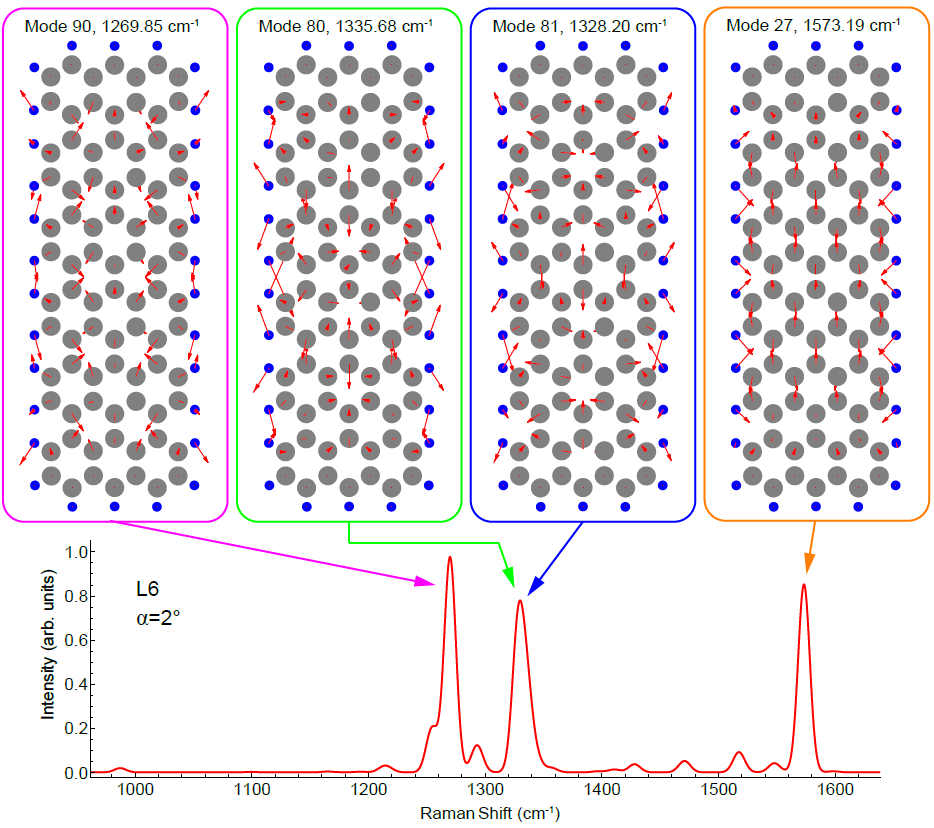
**

**Fig. S14 | Relevant Raman active modes.** Atomic displacements (red arrows) of the normal modes associated to the main peaks for the L6 geometry and $\alpha=2^{\circ}$ bending angle.

**Section IX. Time-Dependent Perturbation Theoretical Model of Coherent Phonon Oscillations**

We use atomic units throughout unless otherwise stated. For a system subject to a time-dependent external potential, the time-dependent wave function $\Psi(t)$ can be expanded in the basis of its field free eigenstates $\psi_{n}$ with eigenvalues $\omega_{n}$,

$\Psi\left( t \right)=\sum_{n} b_{n}(t)\psi_{n}e^{-i\omega_{n}t}$ (1)

As the intensity of the pulses is rather moderate and the sequence of pump and Stokes pulses involves absorption of one photon from the pump pulse and stimulated emission of another photon from the Stokes pulse, one can evaluate the transition amplitudes $b_{n}$ at a sufficiently long time by using second order time-dependent perturbation theory. In this framework, the coefficient that multiplies the initial state remains close to 1 at all times, i.e., $b_{g}\approx1$, and the transition amplitudes to the other states are given, in the frequency domain, by

$b_{ng}\left( \infty\right)=-i\int_{-\infty}^{\infty} d\omega F\left( \omega_{ng}-\omega\right)F\left( \omega\right)\mathcal{M}_{ng}^{\left( 2 \right)}\left( \omega\right)$ (2)

where $F\left( \omega\right)$ is the Fourier transform of the applied external field $F\left( t \right)$

$F\left( \omega\right)=\frac{1}{\sqrt{2\pi}}\int_{-\infty}^{\infty} dtF\left( t \right)\text{e}^{-i\omega t}$ (3)

and $\mathcal{M}_{ng}^{\left( 2 \right)}\left( \omega\right)$ is the usual two-photon matrix element involving a sum over an infinite number of intermediate virtual states. We have added the index *g* to the transition amplitude to indicate that the initial state is the ground state. In our case, $F\left( t \right)$ is the sum of two independent fields, one of them delayed by a time $\tau$ with respect to the other one:

$F\left( t \right)=F_{1}\left( t \right)+F_{2}\left( t-\tau\right)$ (4)

The Fourier transform of $F\left( t \right)$ can be written in terms of the Fourier transforms of $F_{1}$ and $F_{2}$ at zero delay

$F\left( \omega\right)=F_{1}\left( \omega\right)+F_{2}\left( \omega\right)\text{e}^{-i\omega\tau}$ (5)

Substituting the former equation in eq. (2) leads to eight terms representing eight different two-photon paths. In the present experiment, only the path involving absorption of one photon from the pump pulse (hereafter called pulse 1) and stimulated emission from the Stokes pulse (hereafter called pulse 2) will have a significant contribution (the other ones are either negligible in magnitude or contribute to a region of the energy spectrum not accessible in the present experiment). By retaining only this term, eq. (2) reads

$b_{ng}\left( \infty,\tau\right)=-i\int_{0}^{\infty} d\omega F_{2}\left( \omega_{ng}+\omega\right)F_{1}\left( \omega\right)\text{e}^{-i\left( \omega_{ng}+\omega\right)\tau}\mathcal{M}_{ng}^{\left( 2 \right)}\left( \omega\right)$ (6)

where $\omega_{ng}=\omega_{n}-\omega_{g}$ and we have explicitly indicated that the two-photon transition amplitude depends on the delay $\tau$. Notice the sign + in the argument of $F_{2}$ indicating stimulated emission. For simplicity, we will assume that the pulses have perfect gaussian envelopes and have no chirp:

$F_{1}\left( \omega\right)=\frac{1}{\sqrt{2\pi}\sigma_{1}}\text{e}^{-\frac{\left( \omega-\omega_{1} \right)^{2}}{2\sigma_{1}^{2}}}$ (7)

$F_{2}\left( \omega_{ng}+\omega\right)=\frac{1}{\sqrt{2\pi}\sigma_{2}}\text{e}^{-\frac{\left( \omega_{ng}+\omega-\omega_{2} \right)^{2}}{2\sigma_{2}^{2}}}$ (8)

where $\omega_{1}$ and $\omega_{2}$ are the corresponding central frequencies and $\sigma_{1}$ and $\sigma_{2}$the bandwidths. Inserting the two previous equations in eq. (6), we obtain:

$b_{ng}\left( \infty,\tau\right)=-\frac{i}{2\pi\sigma_{1}\sigma_{2}}\int_{0}^{\infty} d\omega\text{e}^{-\frac{\left( \omega_{ng}+\omega-\omega_{2} \right)^{2}}{2\sigma_{2}^{2}}-\frac{\left( \omega-\omega_{1} \right)^{2}}{2\sigma_{1}^{2}}}\text{e}^{-i\left( \omega_{ng}+\omega\right)\tau}\mathcal{M}_{ng}^{\left( 2 \right)}\left( \omega\right)$ (9)

In a more condensed form, the previous equation can be written

$b_{ng}\left( \infty,\tau\right)=-\frac{i}{2\pi\sigma_{1}\sigma_{2}}\mathcal{I}_{ng} \text{e}^{-i\omega_{ng}\tau}$ (10)

where

$\mathcal{I}_{ng}=\int_{0}^{\infty} d\omega\text{e}^{-\alpha\omega^{2}+\beta_{n}\omega+\gamma_{n}}\text{e}^{-i\omega\tau}\mathcal{M}_{ng}^{\left( 2 \right)}\left( \omega\right)$ (11)

with

$\alpha=\frac{1}{2\sigma_{1}^{2}}+\frac{1}{2\sigma_{2}^{2}}$ (12)

$\beta_{n}=-\frac{1}{\sigma_{2}^{2}}\left( \omega_{ng}-\omega_{2} \right)+\frac{1}{\sigma_{1}^{2}}\omega_{1}$ (13)

$\gamma_{n}=-\frac{1}{{2\sigma}_{2}^{2}}\left( \omega_{ng}^{2}+\omega_{2}^{2}-2\omega_{ng}\omega_{2} \right)-\frac{1}{{2\sigma}_{1}^{2}}\omega_{1}^{2}$ (14)

Due to the fact that the chosen photon energies are much larger than the HOMO-LUMO gap in the nanoribbon, evaluation of $\mathcal{M}_{ng}^{\left( 2 \right)}$ for each *n* would require calculating a manifold of intermediate excited electronic states including their corresponding vibrational states. This is not possible in practice. However, since there are many of such possible transitions involving a large variety of electronic states, we can assume that transitions to a given final *n* vibrational state in the ground electronic state will always be allowed. Furthermore, since the region of intermediate states that will effectively contribute to $\mathcal{M}_{ng}^{\left( 2 \right)}(\omega)$is expected to be rather narrow, we will replace the latter quantity by a mean value ${\bar{\mathcal{M}}}_{ng}^{\left( 2 \right)}$ that does not depend on $\omega$. This will allow us to obtain an analytical expression that will be useful to interprete the experimental finding at a qualitative level. In this way, the integral (11) can be expressed in terms of the imaginary error function, leading to

$\mathcal{I}_{ng}=\frac{1}{2}{\bar{\mathcal{M}}}_{ng}^{\left( 2 \right)}\sqrt{\frac{\pi}{\alpha}}\text{e}^{\gamma_{n}}\text{e}^{\frac{1}{4\alpha}\left( \beta_{n}^{2}-\tau^{2}-2\beta_{n}\tau i \right)}\left\{ 1+\text{erf}\left[ \left( \beta_{n}-i\tau\right)/\left( 2\sqrt{\alpha} \right) \right] \right\}$ (15)

The modulus of the erf function approaches −1 when $\sqrt{\beta_{n}^{2}+\tau^{2}}/\left( 2\sqrt{\alpha} \right)\ll0$and to +1 when$\sqrt{\beta_{n}^{2}+\tau^{2}}/\left( 2\sqrt{\alpha} \right)\gg0$. Therefore, in those limits, 1+erf is identical to two times a Heaviside function. As this condition is approximately fulfilled except very close to 0, the above equation can approximately be written as

$\mathcal{I}_{ng}={\bar{\mathcal{M}}}_{ng}^{\left( 2 \right)}\sqrt{\frac{\pi}{\alpha}} \text{e}^{\gamma_{n}}\text{e}^{\frac{1}{4\alpha}\left( \beta_{n}^{2}-\tau^{2}-2\beta_{n}\tau i \right)}$ (16)

This result would also be obtained if the integration was performed between $-\infty$ and $\infty$ instead of between 0 and $\infty$. This would also be a sensible choice since it would imply to assume that the Stokes photon is absorbed and the pump photon is emitted, which is possible for some specific time delays. Inserting equation (16) in eq. (10) and after some algebra, one obtains

$b_{ng}\left( \infty,\tau\right)=-i\mathcal{C}_{n}\text{e}^{-\tau^{2}/\left( 4\alpha\right)}\text{e}^{-i\left( \omega_{ng}+\omega_{n}^{0} \right)\tau}$ (17)

where

$\mathcal{C}_{n}=\frac{{\bar{\mathcal{M}}}_{ng}^{\left( 2 \right)}}{2\sqrt{\pi\alpha}\sigma_{1}\sigma_{2}}\text{e}^{\gamma_{n}}\text{e}^{\beta_{n}^{2}/\left( 4\alpha\right)}$ (18)

and

$\omega_{n}^{0}=\frac{\omega_{1}\sigma_{2}^{2}+\left( \omega_{2}-\omega_{ng} \right)\sigma_{1}^{2}}{\sigma_{1}^{2}+\sigma_{2}^{2}}$ (19)

Therefore, eq. (1) can thus be written as

$\Psi\left( t \right)=\psi_{g}\text{e}^{-i\omega_{g}t}+\sum_{n} b_{ng}\left( \infty,\tau\right)\psi_{n}\text{e}^{-i\omega_{n}t}$ (20)

The probe pulse induces anti-Stokes emission from this coherent superposition. Let us define the state $\phi$ that results from the action of the probe pulse and the subsequent anti-Stokes emission. We will assume that $\left\langle\phi|\psi_{g} \right\rangle=0$, since anti-Stokes emission from $\psi_{g}$ is not possible. Then, the probability of finding $\phi$ at a time $t_{0}$ will be given by

$$\left| <\phi\right|\Psi\left( t_{0} \right)>\left. \right|^{2}=\sum_{n} \left| b_{ng}\left( \infty,\tau\right) \right|^{2}+\sum_{n} \sum_{m\neq n} b_{ng}\left( \infty,\tau\right)b_{mg}^{*}\left( \infty,\tau\right)<\phi\left| \psi_{n}><\phi\right|\psi_{m}>^{*}\text{e}^{-i\omega_{nm}t_{0}}$$

where $\omega_{nm}=\omega_{n}-\omega_{m}$, or more explicitly

$\left| <\phi\right|\Psi\left( t_{0} \right)>\left. \right|^{2}=\sum_{n} \left| \mathcal{C}_{n} \right|^{2}\text{e}^{-\tau^{2}/\left( 2\alpha\right)}\left| <\phi\right|\psi_{n}>\left. \right|^{2}+$

$2\sum_{n} \sum_{m>n} \mathcal{C}_{n}\mathcal{C}_{m}\text{e}^{-\tau^{2}/\left( 2\alpha\right)}\cos\left[ \left( \omega_{n}-\omega_{m}-\frac{\left( \omega_{n}-\omega_{m} \right)\sigma_{1}^{2}}{\sigma_{1}^{2}+\sigma_{2}^{2}} \right)\tau-\omega_{nm}t_{0} \right]\mathcal{R}\left( <\phi\left| \psi_{n}><\phi\right|\psi_{m}>^{*} \right)$ (21)

Therefore, at a given probe time $t_{0}$, the observed anti-Stokes emission leading to state $\phi$ oscillates with a frequency

$f_{\phi}=\omega_{n}-\omega_{m}-\frac{\left( \omega_{n}-\omega_{m} \right)\sigma_{1}^{2}}{\sigma_{1}^{2}+\sigma_{2}^{2}}=\frac{\left( \omega_{n}-\omega_{m} \right)\sigma_{2}^{2}}{\sigma_{1}^{2}+\sigma_{2}^{2}}$ (22)

as a function of the pump-Stokes delay $\tau$. Notice that the amplitude of the oscillations decreases as $\text{e}^{-\tau^{2}/\left( 2\alpha\right)}$. By using the effective pulse durations of the experiment (see Fig. 2b in the main text), this exponential decreases from 1 to 1/e in about 200 fs, in good agreement with the experimental observations. Also notice that, for $\sigma_{1}^{2}\ll\sigma_{2}^{2}$ (in the experiment $\sigma_{1}^{2}\approx0.25\sigma_{2}^{2}$, see Fig. 2b), the frequencies are simply given by $f_{\phi}\approx\omega_{n}-\omega_{m}$, i. e., they correspond to energy differences between phonon frequencies. This is the reason why the frequencies of the oscillations measured in the experiment are close to such values.

We note, however, that, in the experiment, there is not a single observation time $t_{0}$, since the probe pulse is much longer than the Stokes pulse. In reality, the probe pulse probes the generated wave packet in an interval of time $t_{0}$, which runs from $\tau$ up to $\tau+t_{f}$, where $t_{f}$ is approximately half of the probe pulse duration (the second half). Therefore, to reproduce the experimental conditions, one has to integrate Eq. (21) in this time interval. The integrals have the form:

$\int_{\tau}^{\tau+t_{f}} \cos\left[ f\left( \tau\right)-\omega_{nm}t_{0} \right]dt_{0}=\frac{1}{\omega_{nm}}\left( \sin\left[ f\left( \tau\right)-\omega_{nm}\tau\right]-\sin\left[ f\left( \tau\right)-\omega_{nm}\left( \tau+t_{f} \right) \right] \right)$ (23)

which, for small $\omega_{nm}$ (it is always the case), can be approximately written as

$\int_{\tau}^{\tau+t_{f}} \cos\left[ f\left( \tau\right)-\omega_{nm}t_{0} \right]dt_{0}\simeq t_{f}\cos\left[ f\left( \tau\right) \right]$ (24)

Hence, from Eq. (21),

$\frac{1}{t_{f}}\int_{\tau}^{\tau+t_{f}} \left| <\phi\right|\Psi\left( t_{0} \right)>\left. \right|^{2}dt_{0}\simeq\sum_{n} \left| \mathcal{C}_{n} \right|^{2}\text{e}^{-\tau^{2}/\left( 2\alpha\right)}\left| <\phi\right|\psi_{n}>\left. \right|^{2}$

$+2\sum_{n} \sum_{m>n} \mathcal{C}_{n}\mathcal{C}_{m}\text{e}^{-\tau^{2}/\left( 2\alpha\right)}\cos\left[ \left( \omega_{n}-\omega_{m}-\frac{\left( \omega_{n}-\omega_{m} \right)\sigma_{1}^{2}}{\sigma_{1}^{2}+\sigma_{2}^{2}} \right)\tau\right]\mathcal{R}\left( <\phi\left| \psi_{n}><\phi\right|\psi_{m}>^{*} \right)$ (25)

for which the same conclusions as for a well-defined observation time $t_{0}$ can be obtained.

**References**

1 Cai, J. *et al.* Atomically precise bottom-up fabrication of graphene nanoribbons. *Nature* **466**, 470-473 (2010).

2 Cheng, K. N., Lin, Y. H. & Lin, G. R. Single- and double-walled carbon nanotube based saturable absorbers for passive mode-locking of an erbium-doped fiber laser. *Laser Phys.* **23**, 045105 (2013).

3 Puech, P. *et al.* Analyzing the Raman Spectra of Graphenic Carbon Materials from Kerogens to Nanotubes: What Type of Information Can Be Extracted from Defect Bands? *C-J. Carbon Res.* **5**, 69 (2019).

4 Blochl, P. E. Projector Augmented-Wave Method. *Phys. Rev. B* **50**, 17953-17979 (1994).

5 Kresse, G. & Furthmuller, J. Efficiency of ab-initio total energy calculations for metals and semiconductors using a plane-wave basis set. *Comp. Mater. Sci.* **6**, 15-50 (1996).

6 Kresse, G. & Furthmuller, J. Efficient iterative schemes for ab initio total-energy calculations using a plane-wave basis set. *Phys. Rev. B* **54**, 11169-11186 (1996).

7 Kresse, G. & Joubert, D. From ultrasoft pseudopotentials to the projector augmented-wave method. *Phys. Rev. B* **59**, 1758-1775 (1999).

8 Perdew, J. P., Burke, K. & Ernzerhof, M. Generalized gradient approximation made simple. *Phys. Rev. Lett.* **77**, 3865-3868 (1996).

9 Baroni, S. & Resta, R. Ab initio calculation of the macroscopic dielectric constant in silicon. *Phys. Rev. B* **33**, 7017 (1986).

10 Gajdos, M., Hummer, K., Kresse, G., Furthmuller, J. & Bechstedt, F. Linear optical properties in the projector-augmented wave methodology. *Phys. Rev. B* **73,** 045112 (2006).

11 Fonari, A. & Stauffer, S. *vasp_raman.py*. (<https://github.com/raman-sc/VASP/>, 2013).
